# Effect of nutrition education intervention to improve dietary diversity practice and nutritional status of the older people: A cluster randomized controlled trial

**DOI:** 10.1002/fsn3.3667

**Published:** 2023-09-13

**Authors:** Muluneh Shuremu, Kalkidan Hassen Abate, Tefera Belachew

**Affiliations:** ^1^ Department of nutrition and dietetics Jimma University Jimma Ethiopia

**Keywords:** aging population, elderly, Ethiopia, healthy diet, MNA

## Abstract

The growing aging population raises nutrition and health concerns, with malnutrition in the elderly linked to negative health outcomes. Our objective was to implement theory‐based nutritional education interventions to improve the nutritional status of the elderly through diversified dietary practices. A cluster randomized controlled trial was conducted from December 1, 2021, to May 30, 2021, among 782 older persons randomly selected from two urban and 12 semi‐urban areas in southwest Ethiopia. We used Social cognitive theory (SCT) in guiding the nutritional education intervention. Data were collected using an interviewer‐administered questionnaire. The Mini Nutritional Assessment (MNA) tool was used to assess nutritional status, and a qualitative 24‐h eating recall was used to evaluate dietary diversity. Difference‐in‐difference and generalized estimating equation models were used to assess the intervention effect. In total, 720 participants (361 in the intervention group and 359 in the control group) were included for analysis. The mean dietary diversity score differed significantly between the intervention group and the control group (*p* < .001). According to the multivariable generalized estimating equations model, the intervention group was 7.7 times (AOR = 7.746, 95% CI: 5.012, 11.973) more likely to consume a diverse diet than the control group. The nutrition status of the elderly in the intervention group improved significantly at the end of the intervention (*p* < .001). SCT‐based nutritional education interventions can effectively improve healthy eating and nutritional status. For older adults, with its convenient approach and low cost, SCT should be considered an effective and efficient nutritional education approach for behavior change.

## Graphical abstract

Dietary diversity score (DDS). Nutritional status of the elderly people (NSEP). Social cognitive theory (SCT).

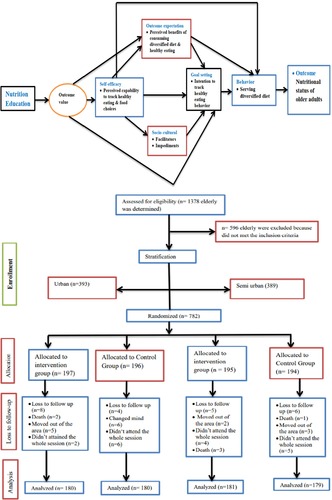

## 1 INTRODUCTION

The elderly population in developing countries is expected to increase over the next 25 years. This could lead to an increased demand for healthcare services and a strain on care resources. To meet these demands, governments will need to invest in healthcare infrastructure and develop new models of care. It represents over 25% of the total population (World Health Organization, 2015).

By 2030, about 75% of the elderly will live in less developed countries. Elderly people in less developed countries have difficult socioeconomic circumstances and their health status is much worse (Boraschi et al., 2010). Proper nutrition is a crucial predictor of effective aging. Poor eating habits in the elderly contribute to the advancement of noncommunicable and chronic illnesses such as type II diabetes, atherosclerosis, coronary heart disease, and malnutrition. Loss of bone density is frequent in the elderly, increasing the risk of osteoporosis (Amarya et al., 2015).

Malnutrition is defined as an overall state of poor nutritional status, including under‐nutrition and overnutrition of macronutrients. Malnutrition in the elderly further increases an individual's risk of developing general poor health or chronic diseases, such as hypertension, type 2 diabetes mellitus, sarcopenia, and cardiovascular disease (Corcoran et al., 2019; Wondiye et al., 2019; Wyka et al., 2012).

The prevalence of malnutrition among elderly people in different countries of the world varies between 10% and 60% (Abate et al., 2020; Konda et al., 2018; Tessfamichael et al., 2014). Malnutrition among older persons is common in Ethiopia, and it remains one of the country's significant public health issues (Abate et al., 2020; Ferede et al., 2022; Kushwaha et al., 2020; Tessfamichael et al., 2014; Wondiye et al., 2019). Studies recommended that further nutrition education intervention is the best strategy to prevent and manage malnutrition among the elderly so that they can achieve a better quality of life and health as they age (Bandura, 1986; Gezahegn et al., 2016; Paul et al., 2022; Poda et al., 2019).

A theoretical framework is the basic foundation upon which evidence‐based interventions are built to achieve successful nutrition interventions (Bandura, 1986). Bandura's Social Cognitive Theory (SCT) (Bandura, 1997b, 2004) is the most commonly used theory in interventions to promote healthy eating behavior. It emphasizes the importance of personal, socio‐environmental and behavioral factors and the interaction between these factors in influencing behavior.

SCT (Bandura, 1997a) provides a conceptual framework that simultaneously addresses psychological, social, and environmental factors related to physical activity. It includes four components: self‐efficacy, outcome expectancies, self‐regulatory behaviors, and barriers such as physical disability (Bandura, 1997b). SCT offers both predictors and principles on how to inform, enable, guide, and motivate people to adapt habits that promote health and reduce those that impair it. Core constructs of this framework include self‐ efficacy, outcome expectations, self‐regulation, and perceived impediments and facilitators of behavior (Bandura, 1997b, 2004).

Older adults who practice healthy eating behaviors may experience an increase in general wellness when socializing (Bandura, 1997a; Hamza et al., 2018). Outcome value precedes intention to make dietary changes, consistent with SCT (Bandura, 1986). Long‐term goals can serve as a general guide and short‐term actions can inform current actions (Bandura, 2004). These factors are assumed to mediate the effect of self‐efficacy on behavior (Hamza et al., 2018).

**FIGURE 1:**
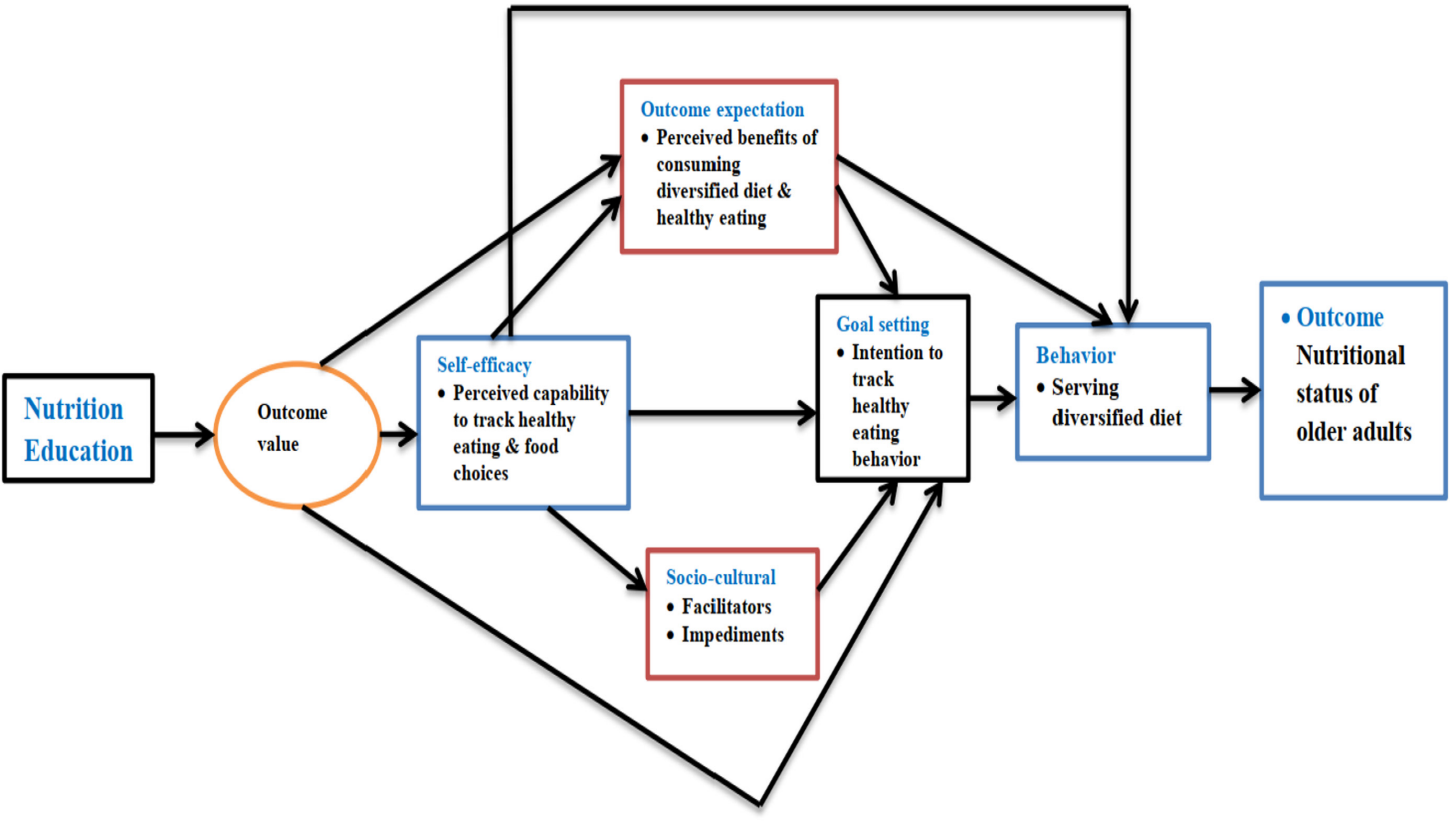
SCT theoretical model for this study. Source: (Bandura, 1997a; Brug et al., 2005).

To the best of our knowledge, this SCT model is the first to mediate diversified dietary practices and healthy eating behaviors among the elderly in Ethiopia; this would make the current study unique. We adapted the model from different sources (Brug et al., 2005; Hamza et al., 2018) to take the advantage of composite constructs of SCT. In Ethiopia, data regarding the effect of nutrition education interventions on the nutritional status of elderly people was scarce. Within this context, the aim of our study was to assess the effectiveness of a health education intervention to improve dietary diversity practices and the nutritional status of older people through improving healthy eating practices using the SCT theoretical model (Bandura, 1997a; Brug et al., 2005) (Figure 1).

## 2 METHODS

### 2.1 Trial design

The effectiveness of a health education intervention to improve older people's dietary diversity practices and nutritional status is investigated in this cluster randomized controlled research (intervention and Control). Participants for a randomized controlled experiment were recruited from southwest Ethiopia's urban and semi‐urban districts between December 2021 and May 2022.

### 2.2 Eligibility criteria

Those eligible to participate in the study were individuals of both sexes, aged 60 years and beyond living in urban and semi‐urban areas of Ilu Aba Bor zone southwest Ethiopia who agreed to participate in the research. The exclusion criteria comprised those who disagreed to give consent; geriatric individuals who were not willing to give an interview; seriously ill people and <60 years old people.

### 2.3 Intervention

In this study, SCT was used to identify the psychosocial processes (behavior mediators) that lead to intervention outcomes. The intervention aimed to improve nutritional behaviors such as taste preferences, attitudes, beliefs, self‐efficacy, health‐related concerns, and body satisfaction (Allison & Keller, 2004; Anderson et al., 2007; Nematollahi & Eslami, 2021) following the completion of the baseline data collection, nutrition education was implemented among intervention groups. The nutrition education program was developed using nutrition education for older adults (Calfas et al., 1997) and other relevant materials devised for nutrition education and counseling guidelines were adopted (Calfas et al., 1997) and modified as a local situation for community‐based nutrition intervention as well as using the findings of a baseline survey. It is easy, interactive, and user‐friendly for all study participants and the research team (Table 1).

**TABLE 1:** Plan for nutrition education intervention for the intervention group.

Weeks	Session title	Time allocated	Description	Teaching Methods	Teaching materials
Week One	Program expectations Introduction to a Healthy Diet for Older Adults and QOL	60 min	This module describes healthy eating for older adults, considering locally available and culturally acceptable food items The health benefits of DD interventions for the elderly The Importance of Nutrition Management for the Elderly How NE improves QOL among the elderly	Brainstorming Role play, group discussion Illustrative and interactive lecture	Leaflets with key messages, pictures Food models
Week Two	Healthy Eating for Successful Living in Older Adults Important nutrients for the elderly (functions and main food sources) Functions and Main Food Sources	120 min	The elderly are educated more about nutrition and how lifestyle changes can promote better health. The focus of this program, which uses behavior change strategies and the My Plate food guide as a framework, is to maintain or improve participants' wellness, specifically heart and bone health, and prevent chronic disease development or progression Eat smart, live strong	Illustrative and interactive lecture Group discussion	Multiple Visual Materials with Pictures to Help the Elderly and Illiterate Patients Understand the Contents
Week Three	Core risk factors affecting the nutritional status of older people	120 min	Behavioral risk factors (tobacco use, harmful alcohol consumption, unhealthy diet [low fruit and vegetable consumption, diet high in salt, insufficient physical activity]) Major biological risk factors (overweight and obesity, raised blood pressure, raised blood glucose, abnormal blood lipids, including raised cholesterol) Physiological risk factors (age‐related changes)	Illustrative, picture‐based, and interactive lecture Group discussion	Leaflets with key messages, pictures
Week Four	Goal setting	60 min	Achieve and maintain moderate physical exercise (30 min) on a daily basis Achieve and maintain normal nutritional status Achieve and maintain healthy eating behavior: high (whole grain, cereals, fruit, vegetables on a daily basis, skimmed milk); low (fat, salt, sweat, or sugar)	Illustrative lecture Role play, demonstration Group discussion Interactive lecture	Leaflets with key messages, pictures
	Outcome expectation	60 min	Dream up for: Better quality of life, normal body composition, self‐confidence, and independence Incentive accomplishments Bright future Active healthy Aging	Brainstorming Illustrative lecture Role play Group discussion Interactive lecture	Leaflets with key messages, pictures
	Self‐efficacy	60 min	Promoting individual capability and self‐confidence to modify desired health behaviors based on self‐monitoring, delivery (+ feedback), and verbal persuasion, knowing how to improve the strength of participants' belief and awareness in their ability to engage in healthier diets to achieve the intended goal despite impediments	Self‐monitoring Illustrative lecture Role play Group discussion Interactive lecture	Leaflets with key messages, pictures
Week Five and onward	Verbal motivation: “You can step towards active healthier aging.” Nutrition counseling	20 min every month for each elderly	Focus on: goal attainment, serving healthy food, limiting unhealthy lifestyles, positive outcome expectations, and maintaining self‐confidence Nutrition counseling is based on the current status of individuals	Home visit, counseling, and discussion	M & E Sheet Handouts and leaflets with core key messages

The Nutrition instructional Package (NTP) consists of five instructional modules and a range of supplemental and relevant educational (Contento, 2011; Nutrition Education for Older Adults, 1985). The communication mechanism was influenced by the context, cultural preferences, and common ways that people absorb and gather information. A number of educational techniques, including group discussions, interactive lectures, role‐plays, active participation, demonstrations, and applicable pictorials, were used to teach the elderly about healthy eating habits.

Based on the best practice guidelines provided by the National Institutes of Health Behavioral Change Consortium, criteria were designed to evaluate the fidelity of the intervention (Bellg et al., 2004; Sheeshka et al., 1993). Checklists to evaluate the intervention design, counselor training, counseling process, acceptance of the intervention, and application of the skills learned from the intervention were part of the criteria (Borrelli, 2011; Sheeshka et al., 1993).

### 2.4 Sample size determination

The sample size was calculated using the G power 3.1.9.4 program with a power of 80% for Fisher's exact test and a precision of 5%. The prevalence of malnutrition among elderly people (P1) was 28.3% (Bandura, 1997b; Brug et al., 2005) and P2 was 13.3%, detecting a 15% difference after the intervention between P1 and P2 (Bandura, 1997a) and the calculated sample size was inflated by the Design effect (DE) assuming 12 clusters in each arm, an inter‐class correlation of 0.007 (Abate et al., 2020; Konda et al., 2018) and cluster size 25 (Bandura, 2004; Rutterford et al., 2015) using the following formula: DE = 1+ (m−1) ICC = 1+ (25–1) 0.007 = 1.168. The total sample size, taking into account a 5% loss to follow‐up, was 368 senior citizens in each arm. Since the elderly who met the criteria were enrolled and cluster randomization was used. Thus, 390 elderly participants in the control group and 392 elderly participants in the intervention group were included in this study.

### 2.5 Randomization

The use of stratified sampling was made. Using a simple random selection procedure, the elderly have been chosen from a number of metropolitan and semi‐urban locations. Two metropolitan areas and twelve semi‐urban areas were chosen from a total of 36 areas. Six clusters were chosen for the intervention based on their proximity, while the remaining six were chosen as the control groups. Six arms were created, each with a buffer zone to prevent information manipulation, for each of the study's six clusters. The remaining six groups were then designated as control groups, and the remaining six clusters were then randomly assigned to an intervention group. The procedure used to determine the rough number of clusters in each arm has already been made public (Rutterford et al., 2015).

### 2.6 Outcomes

The key finding of the study was the nutritional status of each group after the first 6 months, or when the experiment was formally concluded. The primary outcome was explained using, a measure developed by the Nestle Nutrition Institute exclusively for elderly persons (Guigoz, 2006; Kennedy et al., 2007, 2010; Nematollahi & Eslami, 2018). The MNA's overall score allowed for the differentiation between senior individuals who were well‐nourished (scoring 24–30), at risk for malnutrition (score 17–24), and malnourished (score 17).

The trial's secondary outcome required participants to recall every meal they had in the previous 24 h, both inside and outside the home. This was done to determine their dietary variety score. On the day of the interview, unusual consumption (such as that connected with Ramadan and federal holidays) was prohibited. During the reference period, food items that were consumed were assigned a “1,” whereas those that were not consumed were given a “0” (Guigoz, 2006; Swindale & Bilinsky, 2006).

With the most commonly used materials like cups, tablespoons, coconut spoons, food models, and other conventional household measurements, the respondent appraised the subject's consumption. After that, the foods were classified into ten classes using the following criteria: Examples of foods include cereals that are rich in carbohydrates, leafy greens, other fruits and vegetables that are vitamin‐rich, organ meat, fish, eggs, beans, nuts, seeds, dairy products, and fats and oils (Kennedy et al., 2010; Swindale & Bilinsky, 2006).

### 2.7 Data collection

The data were gathered using a pre‐tested, validated, pretested, and structured interviewer‐administered questionnaire that was modified from many sources of research (Contento, 2011; Kennedy et al., 2007, 2010). The questionnaire was translated and administered in local language after translation from English language. The supervisors and data collectors received training on anthropometric techniques and data gathering. To determine the instrument's applicability, a pretest was conducted on 5% of the sample prior the actual data collection and the results were evaluated for any inconsistency and modified accordingly. Cranach's Alpha measure of reliability used and kappa above 0.7 was considered acceptable and all within the acceptable range.

The food security of households was assessed using a method that has been approved for use in other developing countries. The Household Food Insecurity Access Scale (HFIAS), which runs from 0 to 27, was calculated for each participant. Families were categorized into food‐secure, slightly, moderately, or seriously undernourished groups based on their level of food security (Guigoz, 2006).

Principal component analysis was used to build the wealth index. The Household Wealth Index was developed utilizing a variety of variables, including household ownership of fixed assets, services, housing features, and other variables. Quintiles of wealth were then assigned to the latent factor reflecting the wealth index determined by principal component analysis (Arimond et al., 2010).

Anthropometric measurements were made both before and after the intervention. Weight was measured using a digital scale, light clothing, and no shoes (CDC., 2007). The person was measured for height while standing using a portable stadiometer. A calf circumference (CC) of less than 31 cm was given a score of 0 (Smeeth & Woon, 2002; World Health Organization, 2011). The BMI was calculated as weight divided by the square of height (kg/m^2^). Definition provided by the Centre for Disease Control and Prevention (World Health Organization, 2008). Adults with BMIs of 30 kg/m^2^, 25–30 kg/m^2^, and 18.5–25 kg/m^2^ were categorized as obese, overweight, and normal weight, respectively. The waist‐to‐hip ratio was calculated as WC divided by HC. Utilizing a stretch‐resistant tape that could endure 100 g of tension, we measured the hip and waist circumferences. If the two measurements differ by more than 1 cm, more measurements are conducted. If the two measurements are within 1 cm of one another, the average is computed (Bagherniya et al., 2017; CDC, 2007).

The field supervisors provided on‐site assistance to the data collectors every day. Each of their supervisors gathered the completed questionnaires and reviewed them overnight. The supervisors repeatedly conducted shady anthropometric tests and interviews. The study population was defined using summary statistics of means and percentages based on the findings, Sociodemographic characteristics, and other aspects. The data was entered using EpiData version 3.5.1 and exported to SPSS version 22 for analysis. Using Pearson correlation analysis, the relationship between the dietary diversity score, the nutritional state of the elderly, and the SCT components was looked at.

### 2.8 Statistical analysis

The statistical analysis was carried out using SPSS version 22.0. Categorical variables were summarized using numbers and percentages. Quantitative variables were examined for normality and expressed as mean standard deviations (SD). The Student's *t*‐test for independent samples, Mann–Whitney, Chi‐square, and Fisher's exact *t*‐tests were used to compare the baseline characteristics of the intervention and the control groups.

Difference in Differences was used to evaluate the typical treatment effect on the treated by comparing the difference over time in the differences in outcome means in the control and treatment groups. Generalized estimating equations were used to determine how the intervention and control groups differed in their outcomes. The Sociodemographic and economic factors, a few prevalent chronic diseases, food insecurity in households, and lifestyle decisions that can have an impact on older people's nutritional health were all covered by the generalized estimating equations. Each variable in the bivariable was fitted with a multivariable generalized estimating equation with a *p*‐value of .25 and less.

According to the assertion, the adjusted odds ratio (AOR) with matching 95% confidence intervals demonstrated the strength of the relationship. Every analysis took into consideration the Intention to treat (ITT) idea. If a variable's *p*‐value in the multivariable analysis was <.05, it was considered statistically significant.

### 2.9 Ethical considerations

The study was conducted according to the principles of the Helsinki Declaration and the requirements of Good Clinical Practice (Hailemariam et al., 2016). The research protocol was approved by the Institutional Review Board of Jimma University (Ref. No: IHRPGD/419/2019). Written informed consent was secured from each participant before starting the trial.

## 3 RESULTS

### 3.1 Sociodemographic characteristics of the elderly

**FIGURE 2:**
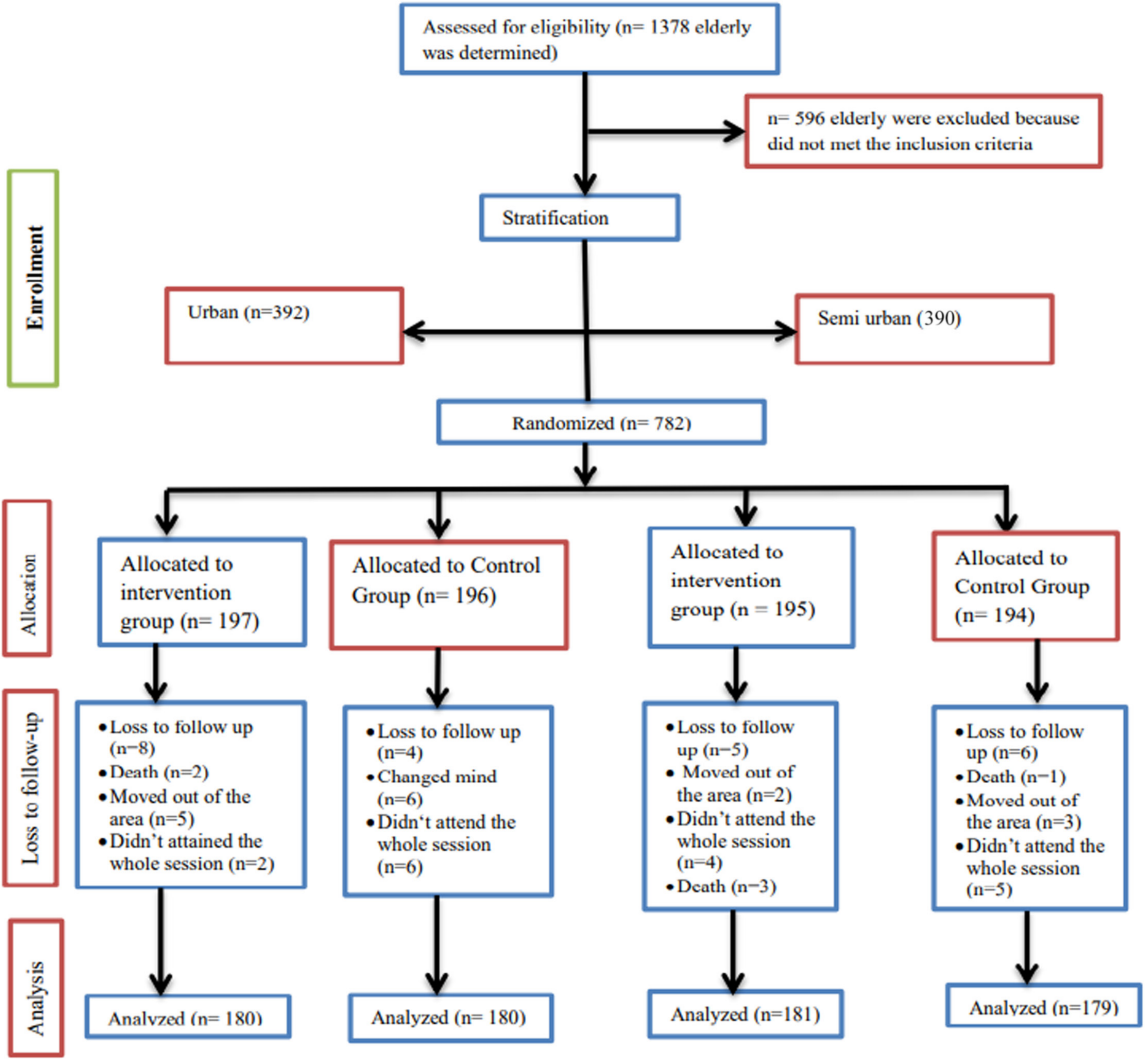
This figure shows the flow of the study participants through the trial according to the criteria recommended in the CONSORT guideline.

Almost 782 individuals (425 women and 357 men) constituted the sample (54.3% and 45.7%, respectively) in total who met the eligibility requirements were randomly assigned to the intervention or control group. Seven hundred twenty (92.1%) of the participants successfully completed the study, including 361 from the education group and 359 from the control group (Figure 2). If a variable's *p*‐value in the multivariable analysis was <.05, it was considered statistically significant.

At baseline, there was no discernible change in the Sociodemographic variables between the intervention and control groups (*p* > .05) (Table 2).

**TABLE 2:** Shows the study participants' baseline Sociodemographic and economic characteristics in the Ilu Aba Bor Zone in southwest Ethiopia (*n* = 782).

Variables	Category	Intervention group	Control group	*p*
		*N* (%)	*N* (%)	
Gender	Female	203 (47.8)	222 (52.2)	.149
	Male	189 (52.9)	168 (47.1)	
Age group (years)	60–69	200 (52.6)	180 (47.4)	.199
	70–79	93 (44.9)	114 (55.1)	
	≥80	99 (50.8)	96 (49.2)	
Residence	Urban	198 (50.4)	195 (49.6)	.887
	Semi Urban	194 (49.9)	195 (50.1)	
Marital status	Single	143 (50.7)	139 (49.3)	.807
	Currently married	249 (49.8)	251 (50.2)	
Religion	Orthodox	159 (52.65)	143 (47.35)	.574
	Muslim	164 (56.75)	125 (43.25)	
	Others	107 (56.0)	84 (44.0)	
Educational status	No formal education	146 (48.2)	157 (51.8)	.661
	Primary education	121 (51.9)	112 (48.1)	
	Secondary and above	94 (51.1)	90 (48.9)	
Respondent's family size	Less than five	200 (58.0)	145 (42.0)	.136
	Five and above	230 (52.6)	207 (47.4)	
Employment status	Government employee	96 (51.0)	92 (49.0)	.378
	Non‐government employee	26 (57.8)	19 (42.2)	
	Self‐employed	181 (58.4)	129 (41.6)	
	Unpaid	127 (53.1)	112 (46.9)	
Wealth quintile	Lowest	100 (52.9)	89 (47.1)	.322
	Second	69 (56.6)	53 (43.4)	
	Middle	51 (47.2)	57 (52.8)	
	Fourth	51 (45.1)	62 (54.9)	
	Highest	89 (47.3)	99 (52.7)	
Household food insecurity	Food secure	141 (47.8)	154 (52.2)	.310
	Food insecure	251 (51.5)	236 (48.5)	

The post‐intervention mean score change for the SCT components is shown in (Table 3). After the intervention, the intervention group's score significantly surpassed the control group's score (*t* (718) = 25.633, *p* < .001). At baseline, the mean outcome value scores in the intervention and control groups were comparable.

**TABLE 3:** Shows the average SCT construct score for the elderly in the Illu Aba Bora Zone of south west Ethiopia (*n* = 720) before and after the intervention.

SCT constructs	SCT constructs a score				Mean difference
	Study period	Intervention (mean ± SD)	Control (mean SD)	*T*‐test	Intervention vs. control
Outcome value	Baseline	27.644 ± 6.57	26.427 ± 5.750	2.643**	1.216
	End line	48.597 ± 1.356	40.711 ± 5.677	25.633***	
Self‐efficacy	Baseline	53.95 ± 7.98	52.81 ± 5.77	2.182*	1.133
	End line	65.55 ± 3.91	60.56 ± 6.66	12.266***	
Outcome expectation	Baseline	27.64 ± 6.57	26.43 ± 5.70	2.643**	1.216
	End line	48.60 ± 1.35	40.71 ± 5.67	25.633***	
Goal setting	Baseline	37.78 ± 8.72	36.58 ± 6.70	2.065*	1.197
	End line	60.72 ± 4.55	55.78 ± 5.99	12.413***	
Socio cultural	Baseline	32.372 ± 3.12	31.438 ± 3.412	3.3826***	0.933
	End line	42.23 ± 3.45	35.32 ± 6.19	18.497***	

*Note*: Values are presented as mean ± SD. *Significant difference between control and intervention (*p* = 0.05). **Significant difference between control and intervention (*p* = .001). ***Significant difference between control and intervention (*p* < .001).

Abbreviation: SD, standard deviation.

At baseline, self‐efficacy did not significantly differ between the two groups; however, as compared to the control group, self‐efficacy significantly increased in the intervention group (*t* (718) = 12.266, *p* < .001). The final outcome expectation significantly outperformed the baseline (718) = 25.633, *p* < .001, just like the baseline outcome expectation. The initial goal‐setting for both groups was equal. By the end of the trial, however, goal‐setting in the intervention group had significantly increased (*t* (718) = 12.413, *p* < .001).

Similar to baseline, the sociocultural construct showed a substantial rise in the intervention group at the end of the study (*t* (718) = 18.497, *p* < .001). Except for self‐efficacy vs. result expectations and sociocultural influences, the connection between SCT components, nutritional status, and dietary diversity score was statistically significant and in a positive direction (Table 4).

**TABLE 4:** Shows the relationships between the SCT components and the DDS and MNA of senior people in the southwest Ethiopian region of Ilu Aba Bor (*n* = 720).

	Outcome expectation	Sociocultural	Goal setting	Self‐efficacy	Outcome value	MNA	IDDS
Outcome expectation	–						
Sociocultural	0.870^a^	–					
Goal setting	0.641^a^	0.800^a^	–				
Self‐efficacy	−0.06	−.0138^a^	0.016	–			
Outcome value	0.639^a^	0.541^a^	0.382^a^	0.335^a^	–		
MNA	0.190^a^	0.220^a^	0.121^a^	0.230^a^	0.179^a^	–	
IDDS	0.168^a^	0.152^a^	0.160^a^	0.118^a^	0.302^a^	0.100^a^	–

Abbreviations: IDDS, Individual Dietary diversity score; MNA, Mini nutritional assessment.

^a^ Correlation is significant at the 0.01 level (2‐tailed).

The results of this study showed that there was no statistically significant difference between the mean MNA at baseline between the intervention and control groups (20.99 vs. 21.29 points) (*p* = .403). However, the end‐line data showed a significant difference between the intervention and control groups in terms of the mean MNA score (24.60 vs. 22.82 points; *p* < .001). Between the intervention and control groups, there was a net mean difference of 2.08 and 0.65 points difference in difference and standard error Table 5 shows that the differences were statistically significant (*p* < .001).

**TABLE 5:** Differences in mean DDS and Mini Nutritional Assessment of the group over the study period in Illu Aba Bor Zone, Southwest Ethiopia (*n* = 720).

Variables	Study period	Intervention group (Mean ± SD)	Control group (Mean ± SD)	df	*T*‐test	*p*	Difference‐in‐difference Mean ± SE, (95% CI) (intervention vs. control)
Mean (±SD) MNA	Baseline	20.99 ± 5.012	21.29 ± 4.534	718	−.837	.403	2.08 ± 0.651 (1.127–2.431)***
	End line	24.60 ± 3.624	22.82 ± 5.142	718	5.363***	<.001	
	Difference (EL‐BL)	3.61 ± 0.612	1.53 ± −.534				
Mean (±SD) DDS	Baseline	4.20 ± 1.45	4.19 ± 1.53	718	0.104	.937	2.21 ± 0.165 (1.998–2.646)***
	End line	6.13 ± 2.35	3.81 ± 2.10	718	14.059***	<.001	
	Difference (EL‐BL)	1.83 ± −.10	−0.38 ± 0.57				

*Note*: Values are presented as mean ± SD, (95% CI). **Significant difference between control and intervention (*p* = .001). ***Significant difference between control and intervention (*p* < .001).

Abbreviations: BL, baseline; EL, End line; SD, standard deviation.

According to this study, the baseline mean dietary variety scores between the intervention and control groups were similar (*t* (718) = 0.104, *p* = .349). However, the end‐line results reveal a significant difference between the intervention group and the control group in terms of mean dietary variety scores (*t* (718) = 14.059, *p* < .001). Following the nutrition education intervention, the independent *t*‐test reveals a statistically significant difference in the mean DDS between the intervention and control group of older persons (DID = 2.20.16) (*p* < .000) (Table 5).

In the multivariable GEE (Table 6), the intervention groups were 7.7 times (AOR = 7.746, 95% CI: 5.012, 11.973) more likely to consume a varied diet than the control group after adjusting for potential confounding variables. A generalized estimating equation was utilized to identify independent parameters linked to the end‐line baseline differences in the variances in mean MNA scores. The factors in the model included sex, educational level, DDS, marital status, family size, occupation, home location, depression, alcohol use, smoking, social support, financial aid, and nutritional education.

**TABLE 6:** Generalized estimating equation illustrating the impact of intervention on the elderly's practice of consuming a variety of food items in the Illu Aba Bor Zone, Southwest Ethiopia (*n* = 720).

Predictors	Nutritional status					
	*β*	SE	*p*	AOR	95% confidence interval	
					Lower	Upper
Threshold	0.977	0.1162	<.001^a^	2.657	2.116	3.336
Time	0.985	0.1317	<.001^a^	2.678	2.061	3.481
DDS Baseline × time = 1	−0.021	0.1570	.895	0.979	0.720	1.332
DDS End line × time = 2	2.026	0.1688	<.001^a^	7.587	5.450	10.562
Intervention group × time	2.047	0.2222	<.001^a^	7.746	5.012	11.973

*Note*: Values are presented as; AOR (95% CI).

Abbreviations: *β* (beta); DDS (dietary diversity score); SE (standard error).

^a^ Significant difference between control and intervention (*p* < .001).

Nutritional education, place of residence, sex, family size, social support, financial support, depression, age, occupational status, food security, DDS, and alcohol consumption were the only independent predictors of MNA among all the other factors. The elderly in the intervention group demonstrated a substantial increase in their nutritional status at the conclusion of the intervention (*β* = 1.119, *p* < .001) after accounting for potential confounders (Table 7).

**TABLE 7:** Generalized estimating equation illustrating the impact of intervention on older people's nutritional status in Illu Aba Bor Zone, Southwest Ethiopia (*n* = 720).

Predictor	Nutritional status					
	*β*	SE	*p*	AOR	95% CI	
					Lower	Upper
Nutritional education						
Time	.477	0.1585	.003	1.611	1.181	2.197
Group	−1.063	0.1846	<.001	0.345	0.241	0.495
Time*group	1.119	0.2302	<.001	3.061	1.950	4.807
Residence area (Ref = semi‐urban)						
Urban	−.273	0.1373	.047	0.761	0.582	0.996
Gender (Ref = male)						
Female	.287	0.0997	.004	1.333	1.096	1.621
Family size (Ref = <5)						
5 and above	−.282	0.1047	.007	0.754	0.614	0.926
Depression (Ref = no)	−.220	0.1045	.035	0.803	0.654	0.985
Wealth index (Ref = highest)						
Lowest	.142	0.1749	.417	1.153	0.818	1.624
Second	−.073	0.1716	.669	.929	0.664	1.301
Medium	.166	0.2411	.491	1.181	0.736	1.894
Fourth	.265	0.2836	.350	1.304	0.748	2.273
Household food security (Ref = food secure)						
Food insecure	−3.397	0.1118	<.001	0.673	0.540	0.837
Social support (Ref = excellent)						
Not at all	−.368	0.1809	.042	0.692	0.485	0.986
Financial support (Ref = excellent)						
Not at all	0.364	0.1590	.022	1.439	1.054	1.966
Smoke (Ref = yes)						
No	0.049	0.1144	.671	1.050	0.839	1.314
DDS (Ref = 4&above food groups)						
<4 food groups	0.032	0.1160	.781	1.033	0.823	1.297
Age (year) (Ref = 60–69)						
70–79	0.636	0.1565	<.001	1.889	1.390	2.568
≥80	0.803	0.1636	<.001	2.232	1.620	3.076
Alcohol drinking (Ref = yes)						
No	0.492	0.1538	.001	1.635	1.210	2.210
Educational status (Ref = secondary & above)						
No formal education	−.020	0.1556	.897	0.980	0.722	1.329
Primary education	0.208	0.1280	.103	1.232	0.958	1.583
Occupational status (Ref = employed)						
Retired	0.467	0.1258	<.001	1.596	1.247	2.042

*Note*: Notably, the model was modified to account for factors such as gender, marital status, drinking, smoking, family size, wealth index, social and financial support, occupation, residential area, education, age, household food security, depression, and DDS, Group * intervention, and interaction.

Abbreviations: *β*, beta (coefficient); AOR, adjusted odds ratio; DDS, dietary diversity score; SE, standard error.

## 4 DISCUSSION

The main purpose of this study was to assess the effectiveness of a health education intervention to improve dietary diversity practices and the nutritional status of older people through a SCT‐guided framework. At baseline, the Sociodemographic parameters and nutritional status of the study participants were comparable. The scores on the SCT components of the intervention groups considerably increased as compared to the baseline score and control group.

The findings of this study indicated that even after a brief intervention, eating behavior modifications and nutrition intake status among female old women were positively impacted by the nutritional education program based on social cognitive theory (Bagherniya et al., 2017). Developing and implementing an educational program based on the social cognitive theory may improve pregnant women's patterns of fruit and vegetable consumption (Seo, 2016).

The results of an Iranian investigation, A Cluster Randomized Controlled Trial (Maryam et al., 2019), which revealed that the SCT domains had altered over time and that there had been a substantial difference between the intervention and control groups, provide weight to this conclusion. In a research of mothers, it was discovered that there was a significant difference between the mean score of SCT structures in the experimental group and the control group before and after the intervention (Sebastian et al., 2021).

The dietary variety score of the structure and the elderly's nutritional state were significantly positively correlated in almost all of the SCT. The enhanced Behavioral mediator changes and their correlation with high DDS and nutritional status demonstrated the efficacy of the intervention in promoting wholesome attitudes, beliefs, values, and expectations of optimal nutrition as we age. Sadly, no interventional investigations on the dietary diversification behavior and nutritional condition of senior Ethiopians were conducted.

This result is comparable to a study from India that found a significant positive link between type II diabetic patients' dietary diversity behavior and all of the Social Cognitive Theory's categories (Sebastian et al., 2021). This may be because people who received nutrition instruction developed more positive attitudes and behavioral control. This suggests that theory‐based nutrition education encourages nutritious eating habits and status.

At the end of the trial, the seniors in the intervention group showed a significant improvement in their nutritional quality after accounting for socio‐demographic parameters such sex, age, residential location, family size, wealth index, marital status, and employment status. Similar studies carried out in Korea (Seo, 2016) shown a beneficial impact of a nutrition education intervention on enhancing the nutritional status of female senior women.

### 4.1 Strengths and limitations

The utilization of intervention strategies, community‐based research, and cluster randomized controlled trial design, and theory‐based nutrition educations are the study's strong points. The following restrictions were recognized, though: All of the conclusions were relied on the senior participants' self‐report, recollection, and honesty in responding to the questions, with the exception of the anthropometric measurement. The lack of comparable references was another drawback.

## 5 CONCLUSION

We found that existing health systems and community institutions may be used to scale up and sustain nutrition education with little financial outlay. The study authors suggested that additional SCT‐based interventional studies be carried out on other behavioral, sociocultural, and environmental facets of the elderly's dietary diversity habit.

## AUTHOR CONTRIBUTIONS

**Muluneh Shuremu:** Conceptualization (lead); data curation (lead); formal analysis (lead); investigation (equal); methodology (lead); project administration (equal); resources (lead); software (lead); supervision (lead); validation (equal); visualization (equal); writing – original draft (lead); writing – review and editing (lead). **Kalkidan Hassen Abate:** Conceptualization (lead); data curation (supporting); formal analysis (supporting); funding acquisition (supporting); investigation (equal); methodology (equal); project administration (equal); resources (equal); software (equal); supervision (supporting); validation (supporting); visualization (supporting); writing – original draft (supporting); writing – review and editing (supporting). **Tefera Belachew:** Conceptualization (equal); data curation (equal); formal analysis (supporting); funding acquisition (supporting); investigation (supporting); methodology (supporting); project administration (supporting); resources (equal); software (equal); supervision (supporting); validation (supporting); visualization (supporting); writing – original draft (supporting); writing – review and editing (supporting).

## FUNDING INFORMATION

The authors have not received any granting support to carry out this research.

## CONFLICT OF INTEREST STATEMENT

The authors declare no conflicts of interest.

## ETHICAL APPROVAL

The Institutional Review Board (IRB) of the Jimma University Institute of Health provided ethical clearance after reviewing the protocol.

## Data Availability

The data that support the findings of this study are available from the corresponding author upon reasonable request.
